# The first two mitochondrial genomes for the genus *Ramaria* reveal mitochondrial genome evolution of *Ramaria* and phylogeny of *Basidiomycota*

**DOI:** 10.1186/s43008-022-00100-7

**Published:** 2022-09-13

**Authors:** Qiang Li, Lijiao Li, Ting Zhang, Peng Xiang, Qian Wu, Wenying Tu, Zhijie Bao, Liang Zou, Cheng Chen

**Affiliations:** 1 Key Laboratory of Coarse Cereal Processing, Ministry of Agriculture and Rural Affairs, School of Food and Biological Engineering, Chengdu University, Chengdu, Sichuan People’s Republic of China; 2 Present Address: Institute of Plant Protection, Sichuan Academy of Agricultural Sciences, Key Laboratory of Integrated Pest Management on Crops in Southwest, Ministry of Agriculture, 20 # Jingjusi Rd, Chengdu, 610066 Sichuan People’s Republic of China

**Keywords:** *Phallomycetidae*, Mitochondrial genome, Intron, Gene rearrangement, Evolution, Phylogenetic analysis

## Abstract

In the present study, we assembled and analyzed the mitogenomes of two *Ramaria* species. The assembled mitogenomes of *Ramaria* cfr. *rubripermanens* and *R. rubella* were circularized, with sizes of 126,497 bp and 143,271 bp, respectively. Comparative mitogenome analysis showed that intron region contributed the most (contribution rate, 43.74%) to the size variations of *Ramaria* mitogenomes. The genetic contents, gene length, tRNAs, and codon usages of the two *Ramaria* mitogenomes varied greatly. In addition, the evolutionary rates of different core protein coding genes (PCGs) in *Phallomycetidae* mitogenomes varied. We detected large-scale gene rearrangements between *Phallomycetidae* mitogenomes, including gene displacement and tRNA doubling. A total of 4499 bp and 7746 bp aligned fragments were detected between the mitochondrial and nuclear genomes of *R.* cfr. *rubripermanens* and *R. rubella*, respectively, indicating possible gene transferring events. We further found frequent intron loss/gain and potential intron transfer events in *Phallomycetidae* mitogenomes during the evolution, and the mitogenomes of *R. rubella* contained a novel intron P44. Phylogenetic analyses using both Bayesian inference (BI) and Maximum Likelihood (ML) methods based on a combined mitochondrial gene dataset obtained an identical and well-supported phylogenetic tree for *Basidiomycota*, wherein *R.* cfr. *rubripermanens* and *Turbinellus floccosus* are sister species. This study served as the first report on mitogenomes from the genus *Ramaria*, which provides a basis for understanding the evolution, genetics, and taxonomy of this important fungal group.

**Supplementary Information:**

The online version contains supplementary material available at 10.1186/s43008-022-00100-7.

## INTRODUCTION

Species from the genus *Ramaria* are usually known as coral fungi because of their colorful and finely branched sporocarps. *Ramaria* species are conspicuous ectomycorrhizal fungi associated with conifers, which are widely distributed in the northern hemisphere (Nouhra et al. 2005). The micro and macro morphological features of *Ramaria* are quite diverse. Some *Ramaria* fruiting bodies are considered to be poisonous, including *Ramaria flavo-brunnescens* and *Ramaria rufescens* (Huang et al. 2009; Perez-Moreno and Ferrera-Cerrato 1995; Scheid et al. 2022), while other *Ramaria* species are edible, such as *Ramaria botrytis*, *Ramaria madagascariensis*, *Ramaria largentii*, *Ramaria flava*, and *Ramaria formosa* (Aprotosoaie et al. 2017; Khaund and Joshi 2014; Liu et al. 2014, 2015). Polysaccharide, sesquiterpenes, and glucan extracted from the basidiocarps of *Ramaria* exhibited antioxidant, antigenotoxic, antitumor, antimicrobial, and immunoenhancing activities (Barros et al. 2008; Bhanja et al. 2013; Centko et al. 2012; Dong et al. 2020; Fu et al. 2022; Li 2017; Liu et al. 2013; Zou et al. 2021). All in all, the accurate identification and classification of *Ramaria* species is very important for the development and utilization of *Ramaria* species.

*Ramaria* species belong to the order *Gomphales* and the subclass *Phallomycetidae*, which occupy a unique phylogenetic position in the higher Basidiomycetes (Nouhra et al. 2005). Taxonomy of the *Gomphales* has traditionally relied upon morphological features now known to be subject to parallel evolution and phenotypic plasticity. Inaccurate classification limits the discovery and utilization of *Ramaria* species. Subsequently, morphological and molecular data (mit-*atp6*-DNA, mit-12S-rDNA, and nuc-25S-rDNA) have been used to infer inter- and intra-specific relationships among *Ramaria* and closely related genera, which reveal that *Gomphales* species are closely related to taxa in the *Geastrales*, *Phallales*, and *Hysterangiales* (Giachini et al. 2010). Mitochondrial genomes (mitogenomes) have been reported to be an effective tool for analyzing the phylogeny of basidiomycetes. Up to now, we have reported four complete mitogenomes from *Phallomycetidae*, including *Turbinellus floccosus* (Cheng et al. 2021), *Sphaerobolus stellatus* (Ye et al. 2020), *Dictyophora indusiate*, and *Phallus echinovolvatus* (Chen et al. 2020a). However, the characteristics of mitogenomes from the genus *Ramaria* are still unknown, which limits our comprehensive understanding of mitochondrial evolutionary pattern and genetics of *Ramaria* species.

Mitochondrial genome is called the ‘second genome’ of eukaryotes, which is considered to be obtained from bacteria through endosymbiosis (Gray et al. 2001). Independent origin, single parent inheritance and several available molecular markers make the mitogenome an effective tool to infer the phylogenetic relationship of species (Abuduaini et al. 2021; Basse 2010). In addition, mitochondrial gene arrangement, variation of conserved genes, dynamic changes of introns, structural variation of tRNA, codon usage and so on also provide rich genetic information for the analysis of species variation and evolution (Fonseca et al. 2021; Ren et al. 2021; Wu et al. 2021a; Zhang et al. 2021a). The mitogenome of fungi has been less studied than that of animals. Mitogenome mutations in animals are considered to be closely related to the occurrence of diseases (Du et al. 2022; Pickett et al. 2022; Zhong et al. 2022). In addition, mitogenomes have also become a powerful tool for analyzing animal systematics and population genetics (Garcia-Souto et al. 2022; Wolfsberger et al. 2022; Zhang et al. 2021b). However, the relationship between fungal mitogenome variation and fungal growth, development and stress response are still unknown. The purpose of obtaining the complete sequence of fungal mitogenome is to further analyze the relationship between fungal mitochondrial sequence mutation and fungal traits. It is reported that the mitogenome of fungi varies greatly in gene arrangement, repeat sequence content, gene number and intron species, even among closely related species (Araujo et al. 2021, Fonseca et al. 2021; Li et al. 2020b; Li et al. 2021b). While most basidiomycetes have been reported to contain a series of core protein coding genes, including *atp6*, *atp8*, *atp9*, cob, *cox1*, *cox2*, *cox3*, *nad1*, *nad2*, *nad3*, *nad4*, *nad4*L, *nad5*, *nad6*, and *rps3* (Li et al. 2021a), the large variations in the content and structure of fungal mitogenomes make it very difficult to obtain a complete fungal mitogenome. Up to now, less than 150 basidiomycete mitogenomes have been published (https://www.ncbi.nlm.nih.gov/genome/browse#!/organelles/), while hundreds of thousands of basidiomycetes have been described in nature. The limited number of available mitogenomes limits our understanding of the genetics and evolution of this diverse fungal group (*Basidiomycota*).

In the present study, the mitogenomes of two *Ramaria* species, including *R.* cfr. *rubripermanens* and *R. rubella*, were assembled and annotated. We also compared with the two *Ramaria* mitogenomes with other mitogenomes from subclass *Phallomycetidae*. The aims of this study are: 1) to reveal the characterizations of the two *Ramaria* mitogenomes; 2) to reveal the variations or conservativeness between *Ramaria* and other *Phallomycetidae* mitogenomes by comparative mitogenomic analysis; 3) to reveal the intron dynamics of *Phallomycetidae* mitogenomes; 4) to understand the phylogenetic status of *Ramaria* in the phylum *Basidiomycota* based on the combined mitochondrial gene set. This study served as the first report on the mitogenome from the genus *Ramaria*, which will promote our understanding of the origin, evolution, and genetics of *Ramaria* species and closely related genera.

## MATERIALS AND METHODS

### Mitogenome assembly and annotation

We assembled the complete mitogenomes of *R.* cfr. *rubripermanens* and *R. rubella* using the raw sequencing data from the Sequence Read Archive (SRA) database under the accession numbers of SRR5801920 and SRR3747475, respectively, which are publically available (Li et al. 2018a, Miyauchi et al. 2020). The specimen of* R*. cfr.* rubripermanens* was that collected in Yunnan used in our previous study (Li et al. 2019a), deposited in the Yunnan Edible Mushroom Research Initiative, Yunnan Agricultural University (number MG17).That of R. rubella was collected by the US Joint Genome Institute (JGI; https://mycocosm.jgi.doe.gov/Ramac1/Ramac1.home.html) for the 1000 Fungal Genome project, and is stored in the USDA Forest Products Laboratory collection, Madison, WI (number UT-36052-T). Quality control steps were conducted to obtained clean reads from the raw sequencing data, which included removing adapter reads using AdapterRemoval v2 (Schubert et al. 2016) and filtering low-quality sequences using ngsShoRT 2.2 (Chen et al. 2014). The two mitogenomes of *Ramaria* were assembled using NOVOPlasty v4.2.1 based on the K-mer size of 29 (Dierckxsens et al. 2017). Gaps between contigs were complemented based on polymerase chain reaction and pyrosequencing. We further obtained the two circularized assembled *Ramaria* mitogenomes. The two complete *Ramaria* mitogenomes were annotated according to previously described methods (Li et al. 2018b). First, the protein-coding genes (PCGs), open reading frames (ORFs), rRNAs, tRNAs, and introns of the two *Ramaria* mitogenomes were annotated according to the results of MFannot (Valach et al. 2014) and MITOS (Bernt et al. 2013) based on the mitochondrial genetic code 4. ORFs longer than 100 aa were selected for further modification or annotation based on the NCBI Open Reading Frame Finder (Coordinators 2017) and BLASTP searches against the NCBI non-redundant protein sequence database (Bleasby and Wootton 1990). Intron and exon boundaries were detected by using exonerate v2.2 (Slater and Birney 2005). The previously annotated tRNA genes in the *Ramaria* mitogenomes were further verified by tRNAscan-SE v1.3.1 software (Lowe and Chan 2016). We drew the physical maps of the two *Ramaria* mitogenomes using OGDraw v1.2 (Lohse et al. 2013).

### Sequence analysis

We calculated base compositions of the two *Ramaria* mitogenomes and other mitogenomes from subclass *Phallomycetidae* using DNASTAR Lasergene v7.1 software (http://www.dnastar.com/). Strand asymmetries of the tested *Phallomycetidae* mitogenomes were calculated according to the following formulas: AT skew = [A − T]/[A + T], and GC skew = [G − C]/[G + C]. We determined codon usages of the two *Ramaria* mitogenomes based on Sequence Manipulation Suite (Stothard 2000). The nonsynonymous (*Ka*) and synonymous (*Ks*) substitution rates for the 15 core PCGs (*atp6, atp8, atp9, cob*, *cox1, cox2, cox3, nad1, nad2, nad3, nad4, nad4L, nad5, nad6,* and *rps3*) in the 6 *Phallomycetidae* mitogenomes were calculated using the DnaSP v6.10.01 (Rozas et al. 2017). The genetic distances between each pair of the 15 core PCGs were calculated using MEGA v6.06 based on the Kimura-2-parameter (K2P) substitution model (Caspermeyer 2016). In addition, we conducted BlastN searches of the two *Ramaria* mitogenomes against themselves (Chen et al. 2015) to find intra-genomic duplications or interspersed repeats in the two *Ramaria* mitogenomes (> 50 bp). Tandem repeats in the two *Ramaria* mitogenomes were determined by using Tandem Repeats Finder (Benson 1999) with default parameters. We also conducted BlastN searches of the two *Ramaria* mitogenomes against their nuclear genomes to identify any gene fragments that transferred between their nuclear and mitochondrial genomes.

### Comparative mitogenomic analysis and intron analysis

We conducted comparative mitogenomic analysis of the 6 *Phallomycetidae* mitogenomes reported to assess variations and conservations between different mitogenomes, which included genome sizes, GC contents, base compositions, codon usage, gene, and intron numbers. We calculated the contribution rate of different regions to the size variation of the two *Ramaria* mitogenomes, which is calculated by the following formula: size difference of region / size different of the entire mitogenome *100%. Introns in *cox1* genes of the 6 *Phallomycetidae* mitogenomes were classified into different position classes (Pcls) according to previously described methods (Ferandon et al. 2010). *Cox1* genes of the 6 *Phallomycetidae* mitogenomes were first aligned with the *cox1* gene of *Ganoderma calidophilum* (Li et al. 2019b), which was used as reference in previous studies (Ye et al. 2020), by using Clustal W (Thompson et al. 1994). The same Pcl was judged by the same insert site in the corresponding reference gene and conservation of sequences, which was named according to their insert site (nt) in the reference gene. Introns belonging to the same Pcl were considered as orthologous intron and usually had a high sequence similarity (Ferandon et al. 2010).

### Phylogenetic analysis

We constructed a phylogenetic tree of 84 *Basidiomycota* mitogenomes using two phylogenetic inference methods based on the combined mitochondrial gene set (14 core PCGs), which included Bayesian inference (BI) method and Maximum Likelihood (ML) methods. The phylogenetic status of *Ramaria* species in the phylum *Basidiomycota* was investigated according to the phylogenetic tree. The phylogenetic tree was constructed based on previously described methods (Huang et al. 2021). *Tuber calosporum* from the phylum *Ascomycota* was used as the outgroup (Li et al. 2020d). Individual mitochondrial genes were first aligned using MAFFT v7.037 (Katoh et al. 2019), and then concatenated into a combined mitochondrial gene dataset using the SequenceMatrix v1.7.8 (Vaidya et al. 2011). Ambiguous regions of these sequences were trimmed. In this dataset, the alignment sequence length of each species used to construct the evolutionary tree reached 21,990 bp. We conducted preliminary partition homogeneity test to detect if there were potential phylogenetic conflicts between different mitochondrial genes. Best-fit models of partitioning schemes and evolution for the combined mitochondrial gene dataset were detected by using PartitionFinder 2.1.1 software (Lanfear et al. 2017). MrBayes v3.2.6 (Ronquist et al. 2012) was used to conduct the BI analysis. Two independent runs with four chains (three heated and one cold) each were conducted simultaneously for 1.0 × 10^7^ generations. Each run was sampled every 1000 generations. We assumed that stationarity had been reached when the estimated sample size (ESS) was greater than 100, and the potential scale reduction factor (PSRF) approached 1.0. The first 50% samples were discarded as burn-in, and the remaining trees were used to calculate Bayesian posterior probabilities (BPP) in a 50% majority-rule consensus tree (Wang et al. 2020b). RAxML v 8.0.0 (Stamatakis 2014) was used to conduct the ML analysis. We assessed Bootstrap values (BS) through an ultrafast bootstrap approach with 1,000 replicates.

### Data availability

The complete mitogenomes of *R.* cfr. *rubripermanens* and *R. rubella* were deposited in the GenBank database (Benson et al. 2018) under the accession numbers OM272988and OM272989, respectively.

## RESULTS

### PCGs in the two *Ramaria* mitogenomes

**Fig. 1:**
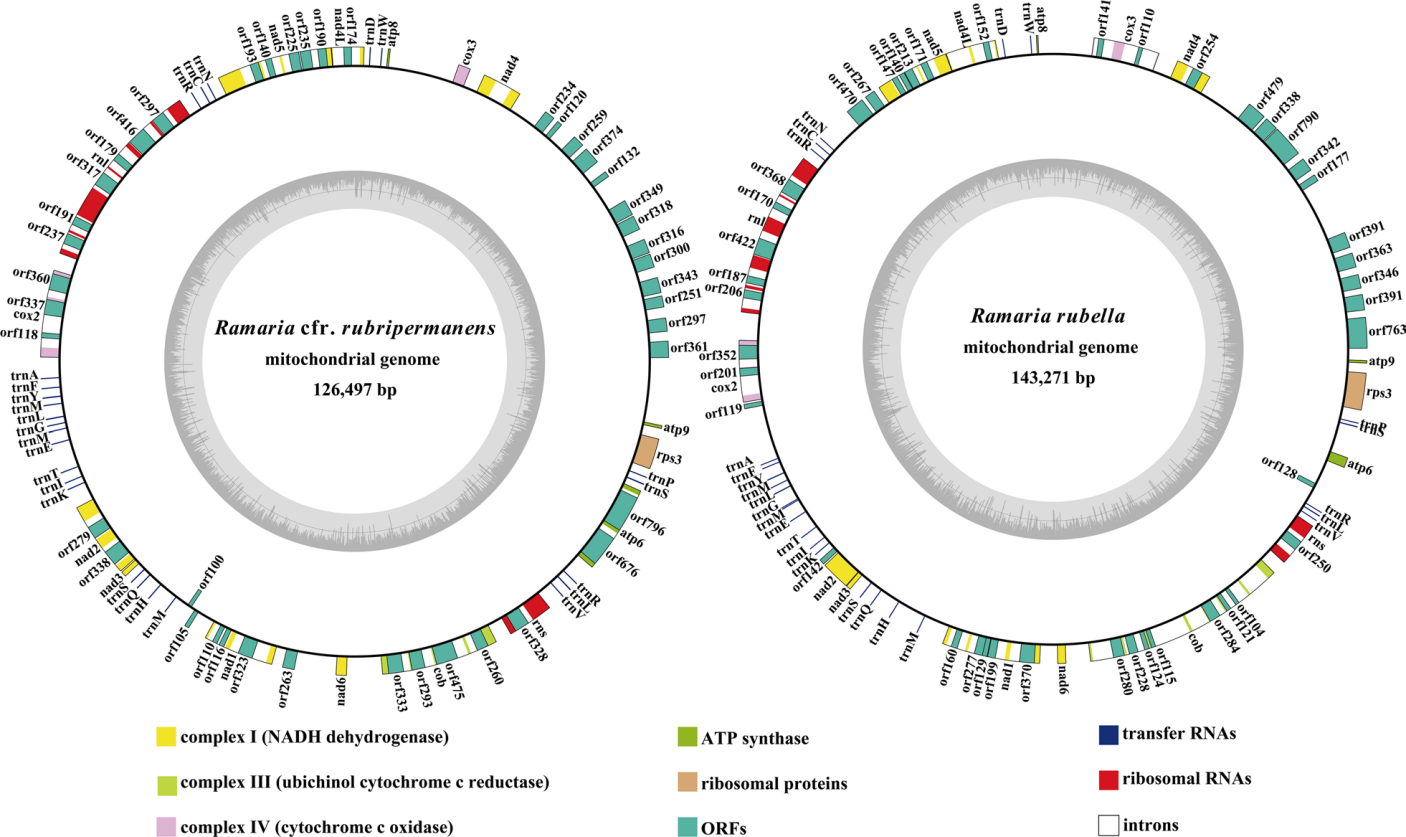
Circular maps of the two *Ramaria* mitochondrial genomes. Genes are represented by different colored blocks. Colored blocks outside each ring indicate that the genes are on the direct strand, while colored blocks within the ring indicates that the genes are located on the reverse strand. The inner grayscale bar graph shows the GC content of the mitochondrial sequences. The circle inside the GC content graph marks the 50% threshold

In the present study, we assembled the two *Ramaria* mitogenomes. The assembled mitogenomes of *R.* cfr. *rubripermanens* and* R. rubella* were circularized, with sizes of 126,497 bp and 143,271 bp, respectively (Fig. 1). The GC content of the *R.* cfr. *rubripermanens* and *R. rubella* mitogenomes were 28.92% and 31.69%, respectively (Additional file 1: Table S1). Both the *Ramaria* mitogenomes had negative AT skews and positive GC skews. A total of 18 and 20 free-stranding PCGs were detected in the mitogenomes of *R.* cfr. *rubripermanens* and *R. rubella*, respectively. Both the *Ramaria* mitogenomes contained a whole set of core PCGs, including *atp6*, *atp8*, *atp9*, *cob*, *cox1*, *cox2*, *cox3*, *nad1*, *nad2*, *nad3*, *nad4*, *nad4L*, *nad5*, *nad6*, and *rps3* (Additional file 1: Table S2). In addition, the mitogenome of *R.* cfr. *rubripermanens* contained 2 non-conserved PCGs encoding proteins with unknown functions and 1 non-conserved PCG encoding a putative GIY-YIG endonuclease. The *R. rubella* mitogenome contained 2 non-conserved PCGs encoding proteins with unknown functions, 1 non-conserved PCG encoding an RNA polymerase and 2 non-conserved PCGs encoding DNA polymerases. The mitogenomes of *R.* cfr. *rubripermanens* and *R. rubella* contained 38 and 37 introns, respectively, which were distributed in *atp6*, *cob*, *cox1*, *cox2*, *cox3*, *nad1*, n*ad2*, *nad4*, *nad4L*, *nad5*, *rns*, and *rnl* genes. Two of the 38 introns from *R.* cfr. *rubripermanens* mitogenome belonged to the group II, 32 belonged to the group I, and 4 were of unknown types. The mitogenome of *R. rubella* contained 3 group II introns, 30 group I introns, and 4 unknown introns. The mitogenomes of *R.* cfr. *rubripermanens* and *R. rubella* also contained 40 and 38 ORFs in introns, which encoded putative LAGLIDADG homing endonucleases, GIY-YIG homing endonucleases, reverse transcriptase/maturase, and proteins with unknown functions.

### RNA genes in the *Ramaria* mitogenomes

**Fig. 2:**
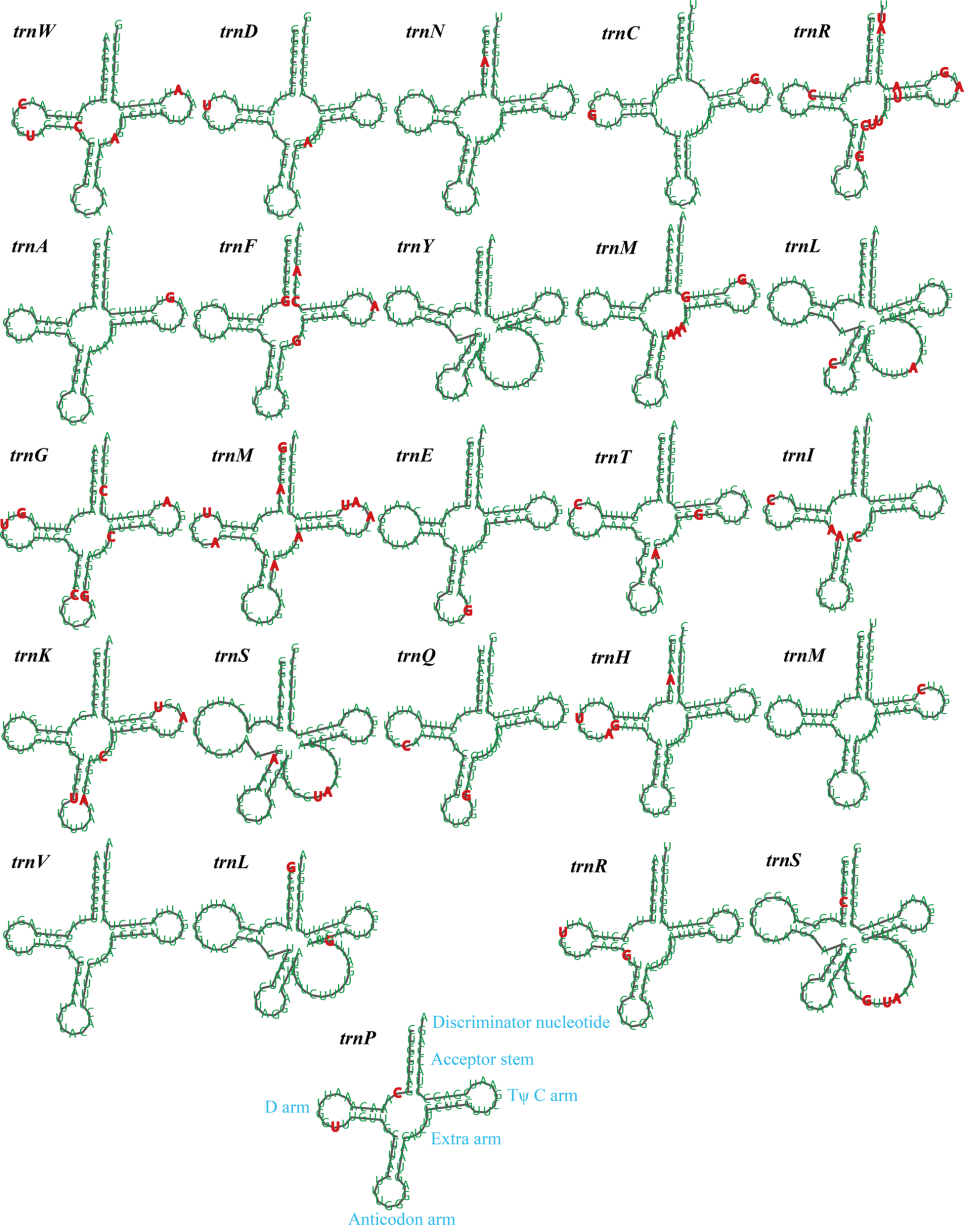
Putative secondary structures of tRNA genes identified in the two *Ramaria* mitochondrial genomes. Residues conserved across the two mitochondrial genomes are shown in green, while variable sites are shown in red. All genes are shown in order of occurrence in the mitochondrial genome of *Ramaria* cfr. *rubripermanens*, starting from *trnW*

We detected two rRNA genes in both the *Ramaria* mitogenomes, which included the small subunit ribosomal RNA (*rns*) and the large subunit ribosomal RNA (*rnl*) (Additional file 1: Table S2). The average lengths of *rns* and *rnl* genes were 1687 bp and 3778 bp, respectively. The *rns* and *rnl* genes of *R. rubella* were 29 bp and 117 bp longer than that of *R.* cfr. *rubripermanens*. Both the *Ramaria* mitogenomes contained 25 tRNA genes, which were folded into classical cloverleaf structures (Fig. 2). The two mitogenomes contained 2 tRNAs with different anticodons coding for arginine, serine, and leucine, and 3 tRNAs with the same anticodons coding for methionine. The size of individual tRNAs ranged from 71 to 88 bp. tRNA genes of sizes > 85 bp, including *trnS*(tga) and *trnL*(tag), contained larger extra arms than other tRNAs, indicating the size variations of tRNAs were mainly due to size variations of extra arms in the *Ramaria* mitogenomes. Of the 25 tRNA genes shared by the two *Ramaria* mitogenomes, 23 contained mutational sites that varied between the two mitogenomes. A total of 83 mutational sites were detected in the 25 tRNA genes between the two *Ramaria* mitogenomes, of which 24.10% variable sites were located on the extra arm, followed by the D arm.

### Mitogenome composition analysis

The mitogenomes of *R.* cfr. *rubripermanens* and *R. rubella* both contained one overlapping nucleotide (Additional file 1: Table S2), which located across the neighboring genes *nad4L* and *nad5* (− 1 bp). The length of intergenic sequences in the two *Ramaria* mitogenomes ranged from 1 to 4480 bp, and the longest intergenic sequence was located between *cox3* and *atp8* in the *R.* cfr. *rubripermanens* mitogenome.

**Fig. 3:**
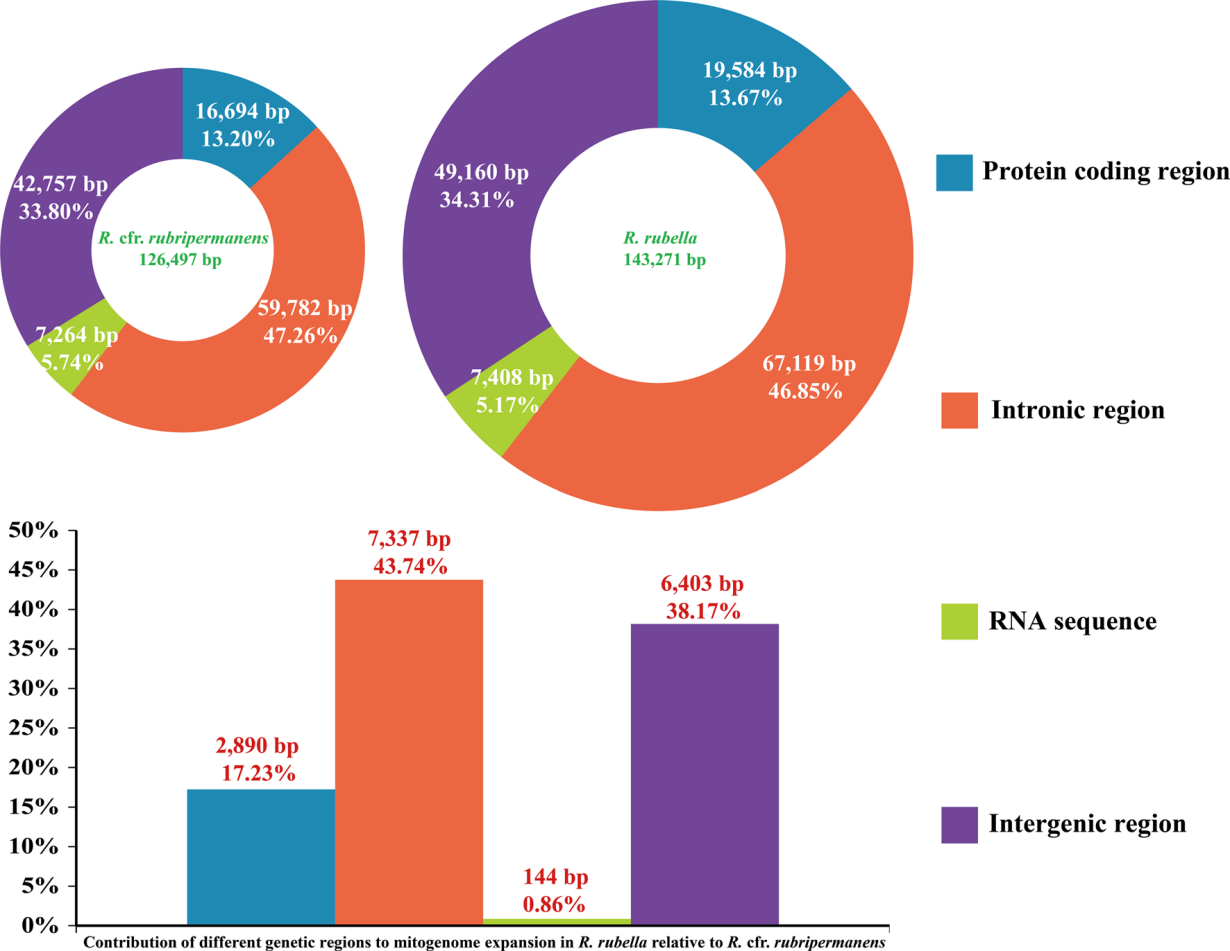
Mitogenome composition of the two *Ramaria* mitochondrial genomes. The bottom panel shows the contribution of different gene regions to the expansion of the *Ramaria rubella* mitogenome. The y-axis represents the contribution rate of different regions to the size variation of the whole mitogenome, which is calculated by the following formula: size difference of region/size different of the entire mitogenome *100%

The intronic regions accounted for the largest proportions of the *R.* cfr. *rubripermanens* and *R. rubella* mitogenomes, which accounted for 47.26% and 46.85% of their mitogenomes, respectively (Fig. 3). Intergenic regions accounted for the second largest proportions of the *R.* cfr. *rubripermanens* and *R. rubella* mitogenomes, accounting for 33.80% and 34.31% of the entire mitogenomes, respectively, which indicated that the two *Ramaria* mitogenomes have relatively relaxed structures. Protein coding regions occupied 13.20–13.67% of the two mitogenomes. RNA coding regions accounted for the smallest proportion of the two mitogenomes (5.17–5.74%). The *R. rubella* mitogenome was 16,774 bp larger than that of *R.* cfr. *rubripermanens*. Intronic regions contributed the most to the size expansion of the *R. rubella* mitogenome, with the contributing rate of 43.74%. Intergenic regions contributed to 38.17% of the size variation, and protein coding regions contributed to 17.23% of the *R. rubella* mitogenome expansion.

### Codon usage analysis

Most of the core PCGs in the 6 *Phallomycetidae* mitogenomes used ATG as start codons, except that the *cox2* gene of *Sphaerobolus stellatus* used GTG, the *nad3* gene of *R.* cfr. *rubripermanens* used TTG, and the *nad6* gene of *S. stellatus* used TTG as start codons (Additional file 1: Table S3). The TAA was the most common used stop codon in the core PCGs of the 6 *Phallomycetidae* mitogenomes, followed by the TAG. The start and stop codons varied greatly between the two *Ramaria* species. The *atp8* and *cob* genes of *R.* cfr. *rubripermanens* used TAG as stop codons, while *R. rubella* used TAA as stop codons. The *nad4* gene of *R. rubella* used TAG as stop codon, while *R.* cfr. *rubripermanens* used TAA as stop codon.

**Fig. 4:**
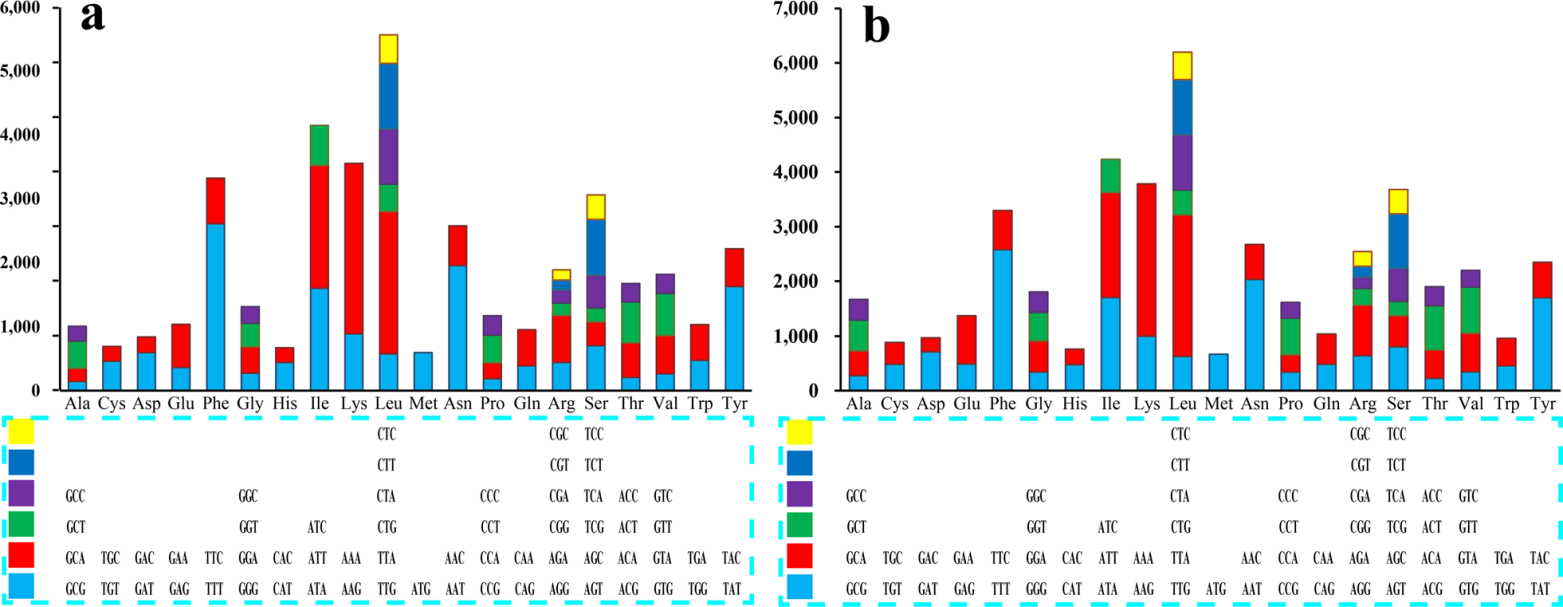
Codon usage of the two *Ramaria* mitochondrial genomes. Frequency of codon usage is plotted on the y-axis. **a**, *Ramaria* cfr. *rubripermanens*; **b**, *Ramaria rubella*

Codon usage analysis indicated that the most frequently used codons in the two *Ramaria* mitogenomes were AAA (for lysine; Lys), TTT (for phenylalanine; Phe), TTA (for leucin; Leu), AAT (for asparagine; Asn), ATT (for isoleucine; Ile), and TAT (for tyrosine; Tyr) (Fig. 4)). The frequent use of A and T in codons contributed to a relative high AT content in the two *Ramaria* mitogenomes (average: 69.70%).

### Repetitive sequences analysis

We conducted BlastN searches of the two *Ramaria* mitogenomes against themselves, and identified 142 and 74 repeat elements in the mitogenomes of *R.* cfr. *rubripermanens* and *R. rubella*, respectively (Additional file 1: Table S4). The length of these repeat sequences ranged from 50 to 413 bp, with pair-wise nucleotide similarities ranging from 76.47 to 100%. The largest repeats were located in the intergenic region between *atp9* and *cox1* gene in the *R.* cfr. *rubripermanens* mitogenome. The largest repeats in the *R. rubella* mitogenome were located in the intergenic region between *trnM* and *trnE*, as well as in intergenic region between *trnE* and *trnT* genes, with each of 100 bp long. Repeat sequences accounted for 9.28% and 3.25% of the *R.* cfr. *rubripermanens* and *R. rubella* mitogenomes, respectively. A total of 37 and 58 tandem repeats were detected in the mitogenomes of *R.* cfr. *rubripermanens* and *R. rubella*, respectively (Additional file 1: Table S5). The longest tandem repeat sequence was detected in the mitogenome of *R.* cfr. *rubripermanens*, comprising 48 bp, which was located in the intergenic region between *cox3* and *atp8*. Tandem repeat sequences accounted for 1.49% and 1.98% of the *R.* cfr. *rubripermanens* and *R. rubella* mitogenomes, respectively.

We further conducted BLAST searches of the two *Ramaria* mitogenomes against their nuclear genomes to identify duplication events between nuclear and mitochondrial genomes. A total of 18 and 22 aligned fragments were detected in the mitogenome of *R.* cfr. *rubripermanens* and *R. rubella*, respectively (Additional file 1: Table S6). The length of these aligned fragments ranged from 100 to 1702 bp, with sequence identities ranging from 76.82 to 100%. The largest aligned fragment was found located in the third intron of *rnl* gene in the *R. rubella* mitogenome. The largest aligned fragment in the *R.* cfr. *rubripermanens* mitogenome (972 bp) was found located in the first exon of *nad2*. A total of 4499 bp and 7746 bp aligned fragment were detected in the mitogenomes of *R.* cfr. *rubripermanens* and *R. rubella*, respectively.

### Evolution and variation of core PCGs

**Fig. 5:**
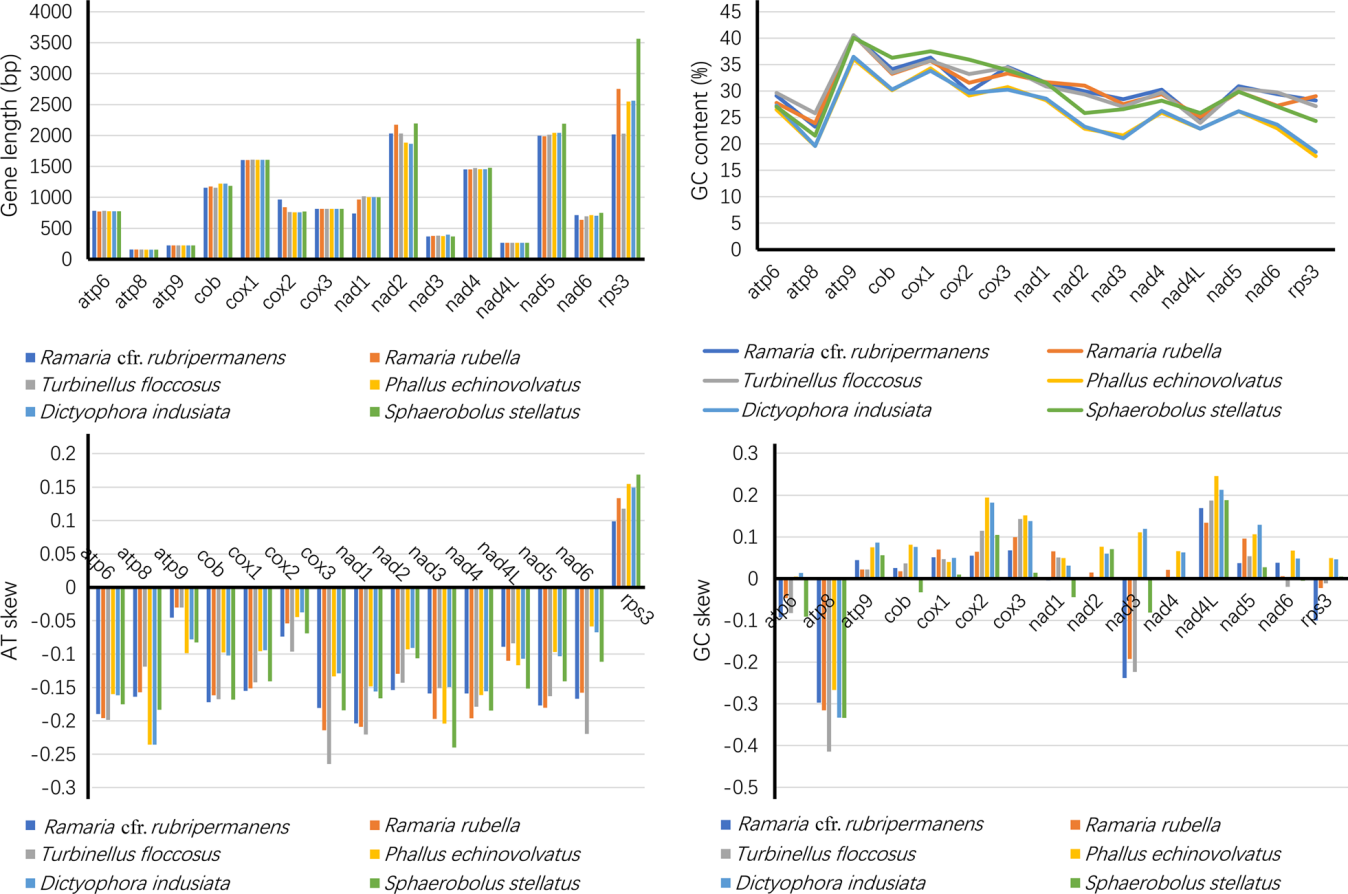
Sequence information of core protein coding genes in the 6 *Phallomycetidae* species

Twelve of the 15 core PCGs showed sequence length variations, with the exceptions of *atp9*, *cox3* and *nad4L*, which had identical gene length among the 6 *Phallomycetidae* species (Fig. 5). The length variation of *rps3* gene was the largest among the 15 core PCGs detected, up to 1545 bp. Among the 15 core PCGs detected, the average GC content of *atp9* was the highest, reaching 39.04%, followed by *cox1* gene, reaching 35.57%. The GC content of *atp8* gene was the lowest, with an average of 22.29%. The GC contents of core PCGs varied among the 6 *Phallomycetidae* species, indicating that there are frequent sequence mutations in core PCGs of *Phallomycetidae* species. Fourteen of the 15 core PCGs contained negative AT skews, and only *rps3* gene contained positive AT skew in the six *Phallomycetidae* species, which showed that most core PCGs tend to evolve in the direction of T-rich rather than A-rich in the leading strand of core PCGs. The GC skews of core PCGs among different *Phallomycetidae* species varied, indicating frequent G/C mutations in the core PCGs.

**Fig. 6:**
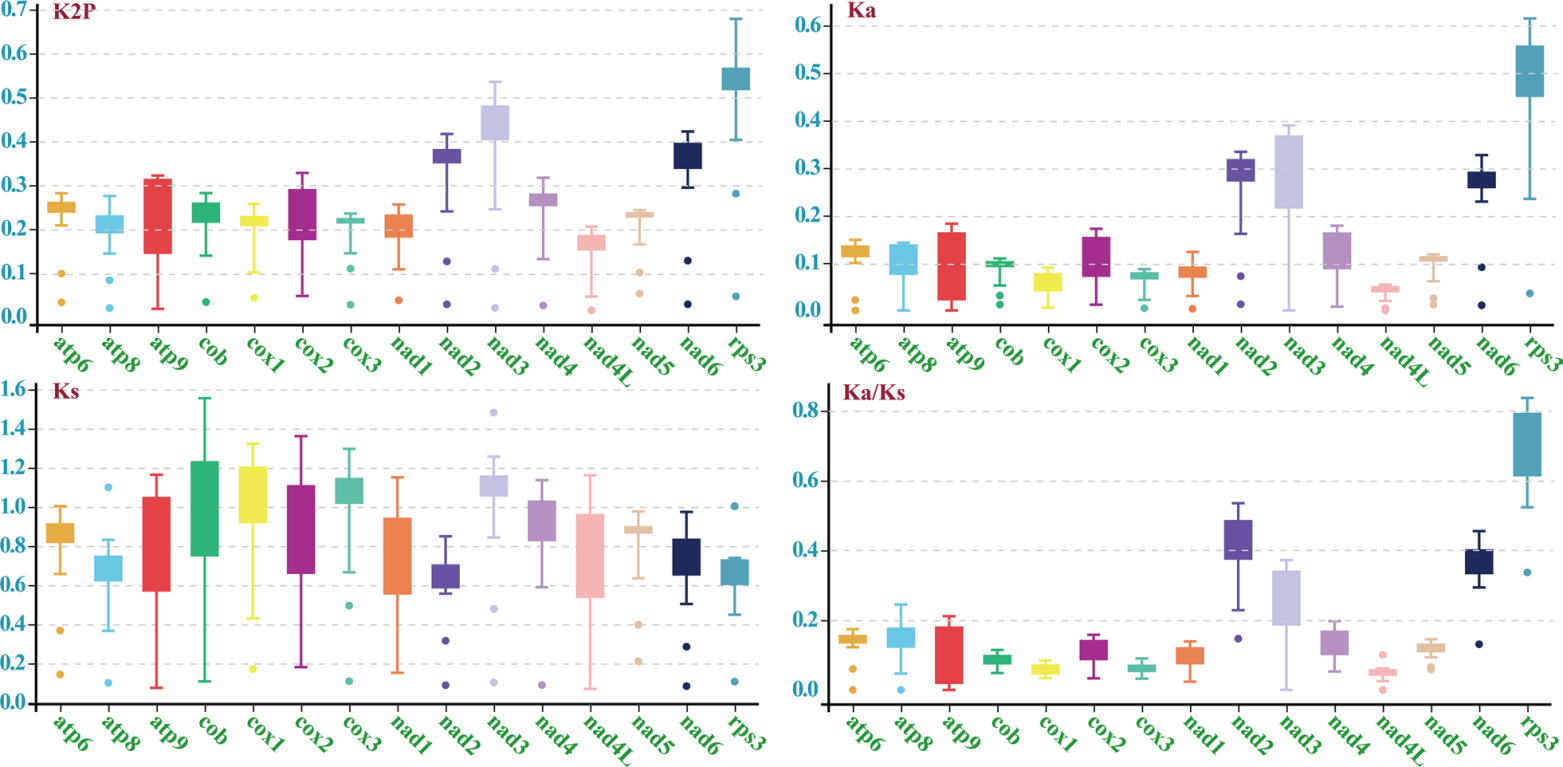
Genetic analysis of the 15 core protein coding genes in the 6 *Phallomycetidae* species. K2P, the Kimura-2-parameter distance; *Ka*, the number of nonsynonymous substitutions per nonsynonymous site; *Ks*, the number of synonymous substitutions per synonymous site

The *rps3* gene was found had the largest average Kimura-2-parameter (K2P) genetic distance between the 6 *Phallomycetidae* species among the 15 core PCGs detected, followed by the *nad3* gene, which showed that these genes differentiated greatly in evolution (Fig. 6). The *nad4L* gene had the smallest average K2P genetic distance between the 6 *Phallomycetidae* species, indicating this gene was highly conserved. The *rps3* gene had the largest non-synonymous substitutions rate (*Ka*) among the 15 core PCGs, followed by the *nad2* and *nad3* genes. While the *nad4L* gene had the smallest *Ka* value. The average synonymous substitution rate (*Ks*) of *nad3* gene was the largest, while that of *nad2* gene was the smallest among the 15 core PCGs. The *Ka/Ks* values for all the 15 core PCGs were < 1, indicating that these genes were subjected to purifying selection.

### Intron dynamics in cox1 genes of *Phallomycetidae*

**Fig. 7:**
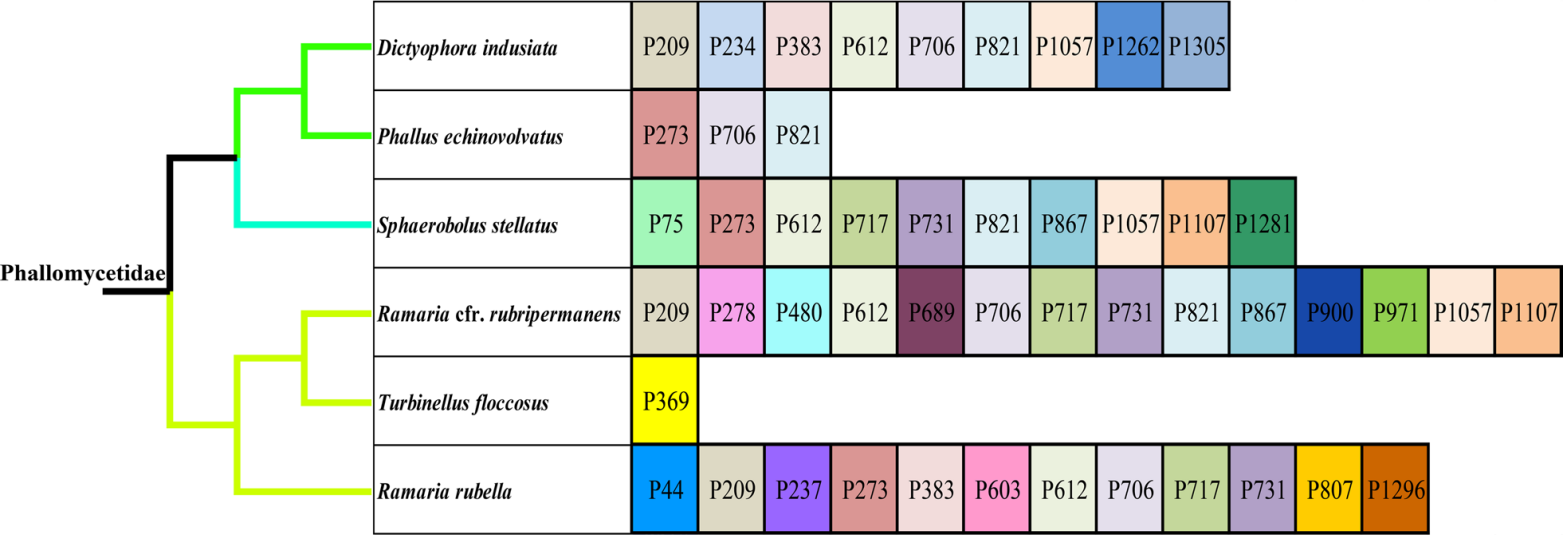
Position class (Pcl) information of *cox1* genes in the 6 *Phallomycetidae* species. Introns in *cox1* genes of the 6 *Phallomycetidae* mitogenomes were classified into different position classes (Pcls) using the *cox1* gene of *Ganoderma calidophilum* as the reference. Each Pcl was constituted by introns inserted at the same position of corresponding *cox1* gene and named according to its insertion site in the aligned corresponding reference sequence (nt). Phylogenetic positions of the 6 *Phallomycetidae* species were established using the Bayesian inference (BI) method and Maximum Likelihood (ML) method based on concatenated mitochondrial genes

A total of 156 introns were detected in the 6 *Phallomycetidae* mitogenomes, which were distributed in *atp6*, *cob*, *cox1*, *cox2*, *cox3*, *nad1*, n*ad2*, *nad4*, *nad4L*, *nad5*, *rns*, and *rnl* genes (Fig. 7). Six of the 156 introns belonged to the group II (Lambowitz and Zimmerly 2004, 2011), 10 were twintrons, and the rest belonged to the group I. We did not detect any putative ORF in 27 of the 156 introns, and about 82.69% introns contained putative ORFs, which encoded putative homing endonuclease, maturases, or reverse transcriptases (Michel et al. 1989). These introns are unevenly distributed in the host gene. *Cox1* gene harbored the largest number of introns, accounting for 31.41% of the total introns, followed by *cob* and *rnl* genes, which harbored 16.03% and 13.46% of the total introns, respectively. We found that introns have a preference for particular host genes, for example, *cox1* gene tends to be rich in introns, which is considered to be independent of the length of host genes (Megarioti and Kouvelis 2020; Michel and Ferat 1995). The *atp8*, *atp9*, *nad3*, and *nad6* did not contain any intron in the 6 *Phallomycetidae* mitogenomes. Intron dynamics in *cox1* gene could significantly affect organization and size of *Phallomycetidae* mitogenomes. We further classified introns in *cox1* genes of the 6 *Phallomycetidae* mitogenomes into different position classes (Pcls) using the *cox1* gene of medical fungus *Ganoderma calidophilum* (Li et al. 2019b) as the reference. Introns belonging to the same Pcl were considered as orthologous introns and had similar intron sequence or structures. The 49 introns in *cox1* genes of the 6 *Phallomycetidae* mitogenomes could be classified into 27 Pcls, which showed the rich diversity of *Phallomycetidae* intron types. The class and number of introns in different *Phallomycetidae* species varied, indicating potential intron loss/gain events in *Phallomycetidae* evolution. The P612, P706, and P821 were the most widely distributed introns (belonging to group I) in *Phallomycetidae*, which were distributed in 4 of the 6 *Phallomycetidae* species. Intron P209, P273, P717, P731, and P1057 were the second common introns (belonging to group I), which could be detected in 3 of the 6 *Phallomycetidae* mitogenomes. However, some rare Pcls could only be detected in one of the 6 *Phallomycetidae* species, such as P237, P369, P807, and P971 (belonging to group I). These rare introns in *Phallomycetidae* could be detected in distantly related species, such as *Pleurotus eryngii* (Li et al. 2018b) and *Laccaria* *bicolor* (Li et al. 2020c) from the order *Agaricales*, *Ganoderma meredithae* (Li et al. 2019b) from order *Polyporales*, and *Rhizopogon vinicolor* from order *Boletales* (Li et al. 2019a), which indicated possible intron transfer events may have occurred in *Phallomycetidae* evolution. A novel intron P44 was detected in *R. rubella*, which belonged to the group II and contained a putative intronic ORF encoding reverse transcriptase. No homologous intron of P44 was detected in other *Basidiomycota* species.

### Gene arrangement and comparative mitogenomic analysis

**Fig. 8:**
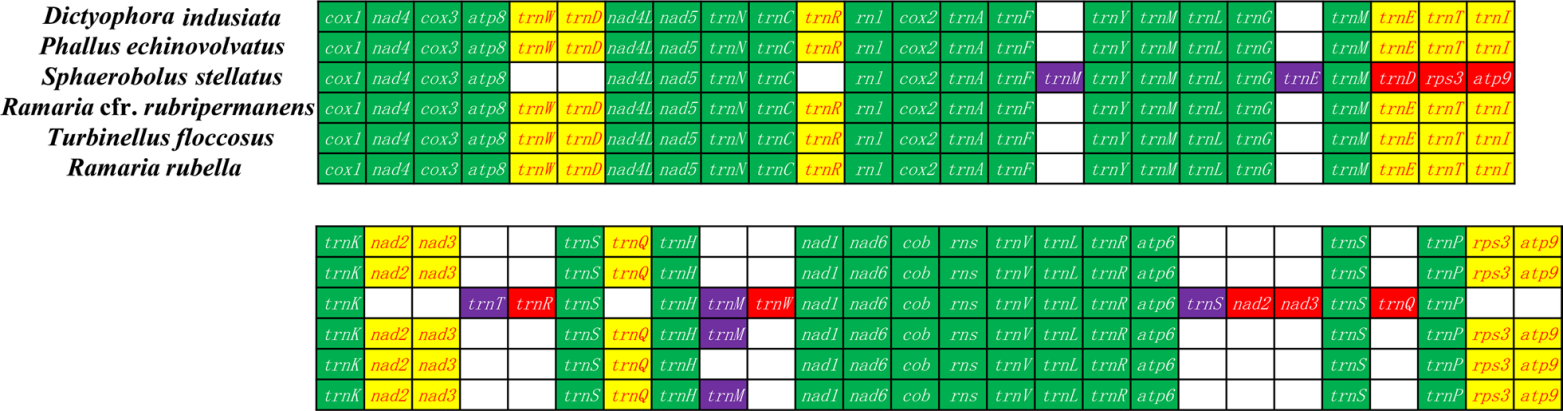
Mitochondrial gene arrangement analyses of the 6 *Phallomycetidae* species. Genes in the green color block indicate that genes from all the six species have identical arrangements; genes in the yellow color block indicate that genes from five of the six species have identical arrangements; genes in the red color block indicate that the gene has been transferred; and genes in the purple color block indicate that the gene has doubled

We further compared mitochondrial gene arrangements of the 6 *Phallomycetidae* species, including PCGs, rRNA genes, and tRNA genes. The results showed that five of the six *Phallomycetidae* species have identical gene arrangement of PCGs and rRNA genes (Fig. 8). Only *Sphaerobolus stellatus* had gene displacement, including *nad2*, *nad3*, *rps3* and *atp9* genes. As far as tRNA genes are concerned, *S. stellatus* had the location transfer of four tRNA genes, including *trnD, trnR, trnW,* and *trnQ*. In addition, four tRNA doubling events have occurred in the *S. stellatus* mitogenome, including *trnM, trnE, trnT*, and *trnS*. The mitogenome of *R.* cfr. *rubripermanens* and* R. rubella* each had one tRNA gene doubling event involving *trnM* gene, respectively.

The size of the 6 *Phallomycetidae* mitogenomes varied, ranging from 50,098 to 152,722 bp, with an average size of 112,345 bp (Additional file 1: Table S1). The two *Ramaria* mitogenomes are larger than the average size of *Phallomycetidae* mitogenomes. The mitogenome of *R. rubella* was the second largest among the 6 *Phallomycetidae* mitogenomes detected, which was only smaller than *Sphaerobolus stellatus* (152,722 bp) from the order *Geastrales*. The GC content of the 6 *Phallomycetidae* mitogenomes ranged from 24.3 to 31.69%, with an average GC content of 27.46%. The two *Ramaria* mitogenomes had a higher GC content than the average value. In addition, there is a large gap (2.77%) in GC content between the two *Ramaria* mitogenomes. Four mitogenomes from *Gomphales* and *Geastrales* had negative AT skews, while the other two mitogenomes from *Phallales* had positive AT skews. Four of the six mitogenomes had positive GC skews, including *R.* cfr. *rubripermanens*,* R. rubella*, *Dictyophora indusiata*, and* Phallus echinovolvatus.* Each mitogenomes contained 16–58 PCGs, and the two *Ramaria* mitogenomes contained the greatest number of PCGs. There were 4–38 introns harboured in the mitogenomes of the 6 *Phallomycetidae* species, and the two *Ramaria* mitogenomes contained the greatest number of introns. All the 6 *Phallomycetidae* mitogenomes contained two rRNA genes. In addition, 24–26 tRNA genes were detected in the 6 *Phallomycetidae* mitogenomes.

### Phylogenetic analysis

**Fig. 9:**
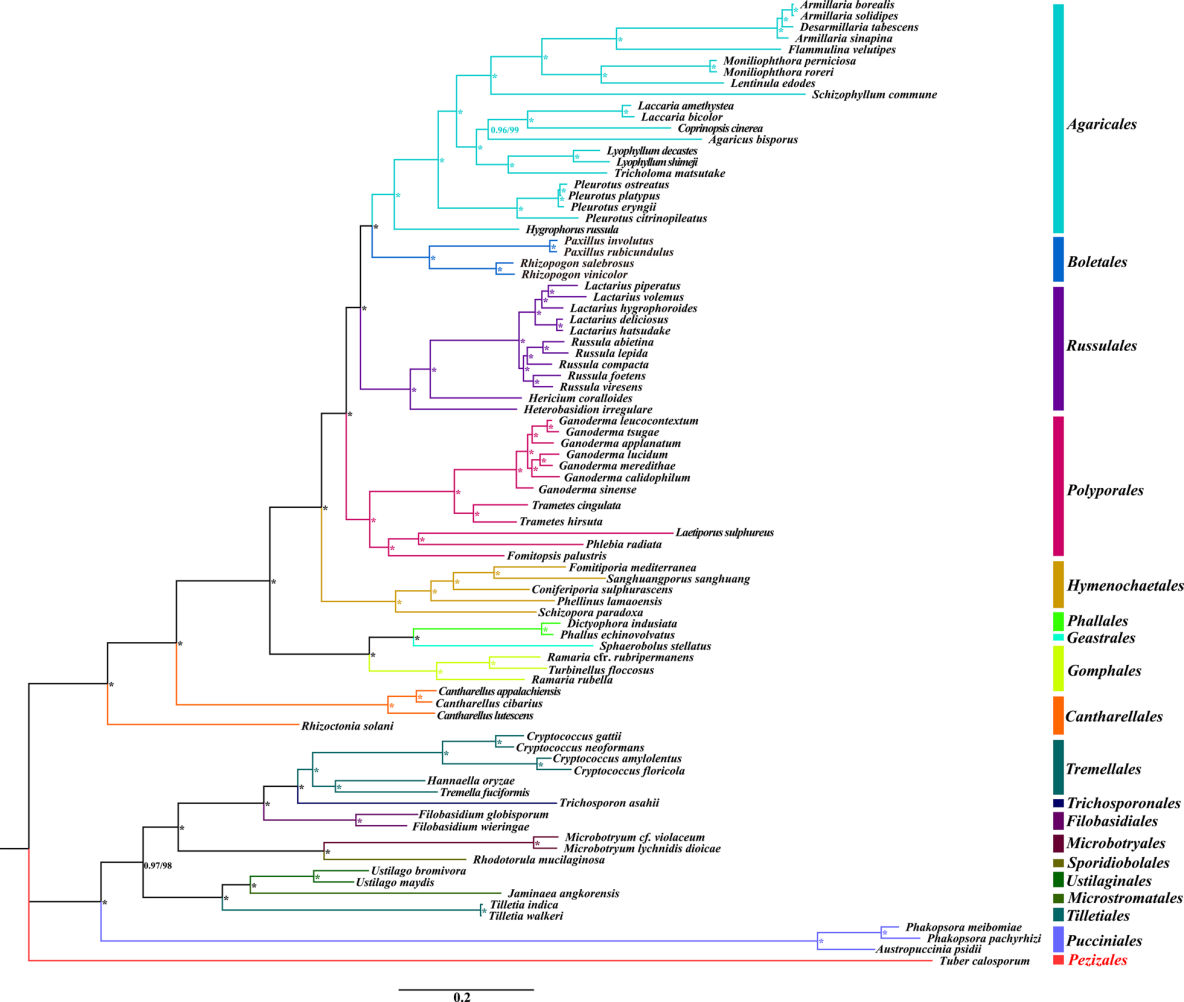
Molecular phylogeny of 84 *Basidiomycota* species based on Bayesian inference (BI) and Maximum Likelihood (ML) analysis of 14 protein coding genes. Support values are Bayesian posterior probabilities (before slash) and bootstrap (BS) values (after slash). The asterisk on the evolutionary clades indicates that the BPP value is 1 and the BS value is 100. Species and NCBI accession numbers for mitogenomes used in the phylogenetic analysis are provided in Additional file 1: Table S7

In the present study, the phylogenetic status of 84 Basidiomycete species was assessed based on combined mitochondrial gene dataset. An identical and well-supported phylogenetic tree was obtained by using both Bayesian inference (BI) and Maximum Likelihood (ML) methods (Fig. 9). All major clades within the phylogenetic tree had a high support value (BPP ≥ 0.96; BS ≥ 98). According to the phylogenetic tree, the 84 *Basidiomycota* species could be divided into 18 major clades, corresponding to the orders *Agaricales*, *Boletales*, *Cantharellales*, *Filobasidiales*, *Geastrales*, *Gomphales*, *Hymenochaetales*, *Microbotryales, Microstromatales, Phallales*, *Polyporales, Pucciniales, Russulales, Sporidiobolales, Tilletiales, Tremellales, Trichosporonales,* and *Ustilaginales* (Additional file 1: Table S7). Phylogenetic analysis indicated that *R.* cfr. *rubripermanens* and *T. floccosus* are sister species, which had a close relationship. The result is consistent with previous studies, indicating the mitogenome was an effective molecular marker for analyze the phylogenetic relationship of basidiomycetes.

## DISCUSSION

Mitogenomes have been reported to play an important role in eukaryotic growth and development, oxidative stress, and environmental response (Cai et al. 2021; Du et al. 2022; Guan et al. 2021). The mitogenome mutation of animals may lead to animal diseases and affect the metabolic level of animals (Chen et al. 2020b). However, as one of the most diverse eukaryotes on earth, the mitogenome structure and variation of basidiomycetes have been less studied (Li et al. 2022; Xu and Wang 2015). Up to now, the complete mitogenome of basidiomycetes available in public databases is less than 0.05% of the described basidiomycete species in nature. It is reported that the mitogenome of basidiomycetes varies greatly in gene content, repeat sequence, genome structure and genome size, which makes it difficult to obtain the complete mitogenome of fungi (Basse 2010; Fonseca et al. 2021; Mendoza et al. 2020). In the family *Phallomycetidae*, the largest mitogenome (152,722 bp) (Ye et al. 2020) is three times larger than the smallest one (50,098 bp) (Chen et al. 2020a). Great genome size variation has also occurred in *Ramaria* species. Previous studies have shown that the size variation of fungal mitogenome is closely related to plasmid-derived genes, repeat accumulation, dynamic changes of introns and variation of intergenic sequence (Boussau et al. 2011; Chen et al. 2021; Wang et al. 2020a; Zubaer et al. 2018). In the present study, we found that introns contribute the most to the mitogenome variation of *Ramaria* species, which is consistent with previous studies (Ye et al. 2020), which showed that introns play an important role in *Phallomycetidae* size variation.

It is reported that the ancestors of eukaryotes obtained mitochondria from bacteria through endosymbiosis (Archibald 2015; Martin et al. 2015; Zimorski et al. 2014). In the long-term evolution process, most mitochondrial genes have been transferred to the nuclear genome, which is convenient for the overall regulation of cells (Adams and Palmer 2003; Barton and Jones 1983). However, some PCGs, tRNA genes and rRNA genes are still retained in the mitogenome (Burki 2016). Among them, a set of core PCGs play an important role in the energy metabolism of eukaryotes. We found that the length, codon usage and base composition of these core PCGs varied greatly between different *Phallomycetidae* species, even among closely related species. The effect of core PCG mutation on fungal growth needs to be further revealed. In addition, different core PCGs have different evolutionary rates and may undergo purifying selection. We also found that 23 of the 25 tRNAs in *Ramaria* species had site variation, and the frequency of mutation may have a certain impact on the growth or stress response of fungi. The mutation of tRNA was considered to be closely related to the efficiency of protein synthesis and may eventually affect the phenotype of eukaryotes (Hayashi et al. 2016; Lin et al. 2021; Povea-Cabello et al. 2020). In the present study, a number of non-conserved PCGs have been detected in *Ramaria* species, which encoded RNA polymerase, DNA polymerase, and proteins with unknown functions. RNA and DNA polymerase are plasmid-derived genes. In some species, they are integrated into the mitogenome, while in others, they exist independently (Wu et al. 2021a, 2021b). The dynamic change of plasmid-derived regions promoted the organization and size variations of fungal mitogenome, and finally makes the fungal mitogenome more diverse and complex (Himmelstrand et al. 2014). In addition, some PCGs with unknown functions exist in *Ramaria* mitogenomes, indicating that the functions of *Ramaria* mitogenomes need to be further analyzed, so as to promote a comprehensive understanding of the function and origin of fungal mitogenome.

Introns are considered as potentially mobile genetic elements in fungal mitogenome, and can affectthe organization and size of fungal mitogenomes (Mukhopadhyay and Hausner 2021). In the present study, we found that introns are closely related to the size variation of mitogenome in subclass *Phallomycetidae*. In basidiomycetes, most mitochondrial introns belong to the group I and only a few introns belong to the group II (Mullineux et al. 2010). Some introns in fungal mitogenome contain intronic ORFs, which can encode putative homing endonuclease, maturases, or reverse transcriptases (Belfort and Lambowitz 2019). Introns can be classified into different position classes according to their insertion sites on the mitochondrial PCGs. Introns belonging to the same Pcls are considered to be orthologous and have high sequence similarities (Cheng et al. 2021; Li et al. 2020a). In this study, we found that the number and types of introns in different *Phallomycetidae* species varied, even among closely related species, indicating that intron loss or gain events have occurred in the evolution of *Phallomycetidae* species. In addition, some rare introns in subclass *Phallomycetidae* were found to exist in a large number in distant species, indicating that there may be potential intron transfer events between them. We also detected a novel intron P44 in *R. rubella* species, which have not been detected in any basidiomycete species reported (Cheng et al. 2021). The origin and evolution of the novel intron P44 need to be further analyzed.

In this study, we also compared the mitochondrial gene arrangement of the 6 *Phallomycetidae* species. Five of the six *Phallomycetidae* species had identical arrangement of PCGs and rRNA genes, which may inherit the gene arrangement from the ancestors of *Phallomycetidae* species. Compared with the gene arrangement of the ancestors of *Phallomycetidae* species, there was a large-scale gene rearrangement in PCGs and tRNA genes of *Sphaerobolus stellatus* (Ye et al. 2020). We also detected the doubling event of tRNAs in *S. stellatus* and the two *Ramaria* species. The mechanism of fungal mitochondrial gene rearrangement has not been fully analyzed. Previous studies have shown that fungal mitochondrial genome rearrangement may be related to the accumulation of repeat sequences (Aguileta et al. 2014). However, we only detected 2.50% repeat elements in the mitogenome of *S. stellatus*, which is lower than the repeat content of the two *Ramaria* species, indicating that the mitochondrial gene rearrangement of *Phallomycetidae* species may be affected by other factors other than repeats. In addition, we detected 4499 bp and 7746 bp aligned fragments between the mitochondrial and nuclear genomes of *R.* cfr. *rubripermanens* and* R. rubella*, respectively, indicating the potential gene fragment transfer events between mitochondrial and nuclear genomes. The coevolution of mitochondrial genome and nuclear genome affects a series of ecological adaptation processes of animals and plants (Bar-Yaacov et al. 2012), which may also be happening in *Ramaria*.

*Ramaria* species is a diverse fungal group. Some *Ramaria* species are edible while others are considered poisonous (Barros et al. 2008; Liu et al. 2014). The misidentification of *Ramaria* species may lead to serious poisoning events. However, some morphological features of *Ramaria* species are easy to overlap, so it is not feasible to classify *Ramaria* species only by morphological features. Mitochondrial genome is considered to be a powerful tool to analyze the phylogenetic relationship of eukaryotes (Luchetti and Plazzi 2021). Compared with traditional multiple molecular markers, which requires multiple PCR and pyrosequencing, mitogenome can provide more genetic information and operate more conveniently. Usually, 15 core PCGs and 2 rRNA genes can be used as molecular markers for phylogeny in mitogenome, resulting in reliable and high support rate of eukaryotic phylogeny (Li et al. 2018b, 2021a; Nie et al. 2019). Although the phylogenetic analysis based on nuclear genome provides more abundant genetic information, the acquisition cost of nuclear genome is high and the amount of data is too large, which limits large-scale access to fungal nuclear genomes. Therefore, phylogeny based on mitochondrial genome is an important choice. *Ramaria* species occupies a unique phylogenetic position in basidiomycetes. While no mitogenome of *Ramaria* species has been reported. In this study, we obtained a highly supported phylogenetic tree based on the combined mitochondrial gene set through two phylogenetic inference methods. Phylogenetic analysis shows that the phylogenetic relationships of *R.* cfr. *rubripermanens*and *T. floccosus* species is closer than that between the two *Ramaria* species, which is consistent with the previous studies based on multiple molecular markers (Giachini et al. 2010), indicating that the mitogenome is a reliable tool for analyzing the phylogenetic relationship of *Phallomycetidae* species. More mitogenomes of *Phallomycetidae* species need to be obtained to understand the population genetics and phylogeny of *Ramaria*.

## CONCLUSIONS

In this study, we obtained two novel mitogenomes from the *Ramaria* genus. The complete mitogenomes of *R.* cfr. *rubripermanens* and *R. rubella* showed large size variations, and the intron region contributed the most to the size variation of two mitogenomes (contribution rate, 43.74%). We further detected large variations in genetic contents, gene length, tRNAs, and codon usages of the two *Ramaria* mitogenomes. Comparative genomic analysis detected large-scale gene rearrangements between *Phallomycetidae* mitogenomes, including gene displacement and tRNA doubling. We first found possible gene transferring events between the mitochondrial and nuclear genomes of the two *Ramaria* species. The *Phallomycetidae* mitogenomes have been experienced frequent intron loss/gain and potential intron transfer events during the evolution, and a novel intron P44 was found in the *R. rubella* mitogenome. Phylogenetic analyses showed that phylogenetic relationships of *R.* cfr. *rubripermanens* and *T. floccosus* species is closer than that between the two *Ramaria* species. The present study provides the first record of mitogenomes from this genus *Ramaria*, thus providing a foundation for studying the evolution, genetics, and taxonomy of this important genus and related fungal group.

## Supplementary Information

**Additional file 1**.** Table S1**. Comparison on mitogenomes among six Phallomycetidae species.** Table S2**. Characterization and annotation of the two* Ramaria* mitogenomes.** Table S3**. Start and stop codons analysis of 6 species from Phallomycetidae.** Table S4**. Local BLAST analysis of the two* Ramaria* mitogenomes against themselves.** Table S5** Tandem repeats detected in the two* Ramaria* mitogenomes.** Table S6**. Aligned fragments between mitogenomes and nuclear genomes of the two Ramaria species.** Table S7**. Species information used for phylogenetic analysis in this study.

## Data Availability

All data generated or analyzed during this study are included in this published article [and its Additional files].

## Abbreviations

Mitogenome: Mitochondrial genome
PCG: Protein-coding gene
Pcls: Position classes
*Ks*: Synonymous substitution rates
*Ka*: Nonsynonymous substitution rates
BI: Bayesian inference
ML: Maximum Likelihood
